# A Meta-Analysis of Short-Term Outcomes After Laparoscopic Lavage Versus Colonic Resection in the Treatment of Perforated Diverticulitis

**DOI:** 10.7759/cureus.34953

**Published:** 2023-02-14

**Authors:** Jonathan Tiong, Rufi Chen, Sachin Phakey, Ned Abraham

**Affiliations:** 1 General Surgery, Monash Health, Melbourne, AUS; 2 General Surgery, Royal Melbourne Hospital, Melbourne, AUS; 3 Faculty of Medicine, University of New South Wales Australia, Coffs Harbour, AUS; 4 Department of Colorectal Surgery, Baringa Private Hospital, Coffs Harbour, AUS

**Keywords:** colonic resection, laparoscopic lavage, hinchey classification, perforated diverticulitis, complicated diverticulitis, sigmoid diverticulitis

## Abstract

The management of perforated non-faeculent diverticulitis has traditionally involved performing a colonic resection (CR). Laparoscopic lavage (LL) has emerged as a less invasive alternative in recent years. The aim of this meta-analysis was to assess the role of LL in the surgical treatment of perforated non-faeculent diverticulitis. To that end, we conducted a search on Embase, Medline, and Cochrane databases for comparative studies in the English language published till June 2021 [PROSPERO (CRD42021269410)]. The risk of bias was assessed using the revised Cochrane risk-of-bias tool for randomised trials (RoB 2)* *and the methodological index for non-randomised studies (MINORS). Data were analysed using Cochrane RevMan. Pooled odds ratio (POR) and cumulative weighted ratios (CWR) were calculated.

A total of 13 studies involving 1061 patients were found eligible, including seven studies based on three randomised control trials (RCTs). LL was associated with a reduced risk of wound infection, stoma formation, and need for further surgery by 77% [POR: 0.23, 95% confidence interval (CI): 0.07-0.74], 83% (POR: 0.17, 95% CI: 0.05-0.56), and 53% (POR: 0.47, 95% CI: 0.23-0.97) respectively. Duration of surgery and hospitalisation was reduced by 54% and 43% respectively. However, LL was associated with higher rates of unplanned reoperations (POR: 2.05, 95% CI: 1.22-3.42), recurrence (POR: 9.47, 95% CI: 3.24-27.67), and peritonitis (POR: 8.92, 95% CI: 2.71-29.33). No differences in mortality or readmission rates were observed.

LL in Hinchey III diverticulitis lowers the incidence of stoma formation and overall reoperations without an increase in mortality but at the cost of higher recurrence rates and peritonitis. A limitation of this study was the inclusion of non-RCTs. An elective resection should be considered after LL. Guidelines for surgical techniques in LL need to be standardised.

## Introduction and background

Complications of diverticular disease are one of the most common gastrointestinal causes of hospitalisation in the United States [1]. There is a consensus that acute diverticulitis with abscess or phlegmon formation should be treated initially with intravenous antibiotics with or without percutaneous drainage [2]. Faeculent diverticulitis requires colonic resection (CR) with or without a primary anastomosis, often with a “covering” ileostomy [3].

However, the surgical management of perforated purulent diverticulitis (Hinchey III) remains a matter of controversy. Traditionally, the treatment has been CR as described above. Emergency laparoscopic lavage (LL) for perforated diverticulitis was first described in 1996 and has since been proposed as an alternative to emergency CR [4], with the impetus being the avoidance of major morbidities such as an anastomotic leak or stoma formation. Proponents of LL point to the results of several noncomparative studies that suggest that LL may be a suitable option for patients with diffuse purulent, non-faeculent peritonitis, with no visible free perforation [5,6]. Complication rates were reportedly lower than in the case of CR, with no increase in mortality rates. In addition, the European Society of Coloproctology guidelines recommend LL as a feasible approach to patients with Hinchey III peritonitis [7]. However, the American Society of Colon and Rectal Surgeons recommends CR over LL as the preferred treatment of choice [3].

The aim of this meta-analysis was to compare the outcomes of LL with those of CR among patients with perforated purulent diverticulitis requiring surgical intervention.

## Review

Materials and methods

The meta-analysis was performed in accordance with the Preferred Reporting Items for Systematic Reviews and Meta-Analyses (PRISMA) guidelines. The protocol for this meta-analysis is available in PROSPERO (CRD42021269410): https://www.crd.york.ac.uk/prospero/display_record.php?RecordID=269410.

Literature Search Strategy and Selection Criteria

An electronic search was performed using Embase (1974 to June 2021), Medline (1946 to June 2021) and Cochrane databases to identify studies in English comparing LL with CR for acute perforated diverticulitis. Medical Subject Headings (MESH) and keywords, such as “diverticulitis, colonic” (MESH), “diverticulum” (MESH), “perforated”, “diverticulitis”, “therapeutic irrigation” (MESH), “laparoscopy” (MESH), “lavage”, “colectomy” (MESH) and “resection”, in combination with Boolean operators AND or OR were used. A manual search based on the reference lists of obtained articles and through Google Scholar was also performed.

Two authors (RC and NA) independently examined the abstracts of the initially selected studies to determine eligibility. Publications were included if the study were randomised control trials (RCTs) or non-randomised comparative studies (NRCS) involving LL versus CR in patients with acute non-faeculent diverticulitis requiring emergent surgical intervention. CR included procedures performed laparoscopically or using an open approach. Studies that did not compare outcomes of LL versus CR were excluded from the analysis.

Quality Assessment

The quality of the included trials was assessed independently by two authors (SP and JT). RCTs were appraised using the revised Cochrane risk-of-bias tool for randomised trials (RoB 2) [8]. The methodological index for non-randomised studies (MINORS) was used to assess NRCSs [9]. Disagreements were resolved by discussion and consensus.

Data Extraction

Data from the included studies were retrieved by two investigators (SP and RC). Data were entered into a Microsft Excel® spreadsheet. Extracted data included patient demographics, duration of surgery, operative and postoperative complications, mortality rates, length of hospitalisation, ICU admission, stoma and readmission rates, and quality-of-life scores. The recorded surgical postoperative complications included recurrence, peritonitis (generalised or contained), wound infection, and systemic complications. Supplemental data were retrieved and included when available.

Statistical Analysis

Statistical analysis was performed using Cochrane RevMan (version 5.4.1). Pooled odds ratio (POR) with 95% confidence intervals (CI) were calculated for dichotomous variables. Where Cochran’s Q test showed significant heterogeneity, pooled outcome measures were determined using the random-effects model as described by DerSimonian and Laird. Cumulative weighted ratios (CWR) were used for continuous variables. The calculation of CWR has been described elsewhere [10]. Briefly, whether mean or median values for continuous variables were recorded, the differences between the two treatment groups were calculated as ratios. The ratios were given weights depending on sample size, and CWRs from all studies were calculated. A p-value <0.05 was considered statistically significant. Subgroup analysis was conducted based on study design factors.

Results

Description of Eligible Studies

**Figure 1:**
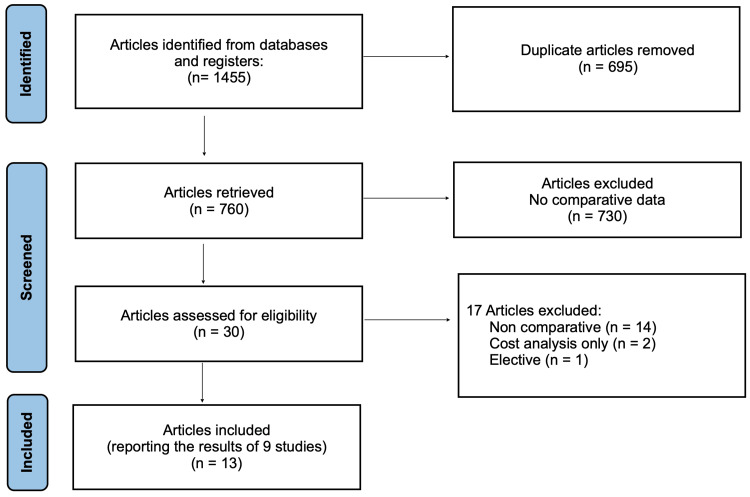
PRISMA flow diagram depicting the literature search PRISMA: Preferred Reporting Items for Systematic Reviews and Meta-Analyses

The PRISMA flow diagram of the literature search is shown in Figure 1. The electronic database search yielded 1,455 publications. A total of 32 articles were retrieved and considered for the meta-analysis. Seventeen studies were excluded as they did not present comparative data for LL and CR. Of the 13 studies included in the meta-analysis [11-23], seven examined data from three RCTs at different time points of follow-up, and six were NRCSs (Table 1).

**Table 1:** Articles included in the meta-analysis comparing outcomes following laparoscopic lavage versus colonic resection for perforated non-faeculent diverticulitis CR: colonic resection; DILALA: Diverticulitis–Laparoscopic Lavage; HP: Hartmann’s procedure; JVUH: Jean Verdier University Hospital; LADIES: Laparoscopic Peritoneal Lavage or Resection for Generalised Peritonitis for Perforated Diverticulitis; LL: laparoscopic lavage; ^n^CR: colonic resection sample size; ^n^LL: laparoscopic lavage sample size; PRA: primary resection anastomosis with or without diverting loop ileostomy; SCANDIV: Scandinavian Diverticulitis trial; TEICP: Texas Endosurgery Institute Colorectal Procedures database

Study	Country	Trial name/data source	Study design	Study period	n_LL_	n_CR_	CR intervention
Randomised controlled trials (7)							
Azhar et al., 2021 [11]	Sweden, Norway	SCANDIV	Randomised controlled trial (21 centres)	Feb 2010–Jun 2014	73	69	Open or laparoscopic, HP or PRA
Kohl et al., 2018 [16]	Sweden, Denmark	DILALA	Randomised controlled trial (9 centres)	Feb 2010–Feb 2014	43	40	Open HP
Schultz et al., 2017 [12]	Sweden, Norway	SCANDIV	Randomised controlled trial (21 centres)	Feb 2010–Jun 2014	74	70	Open or laparoscopic, HP or PRA
Angenete et al., 2016 [14]	Sweden, Denmark	DILALA	Randomised controlled trial (9 centres)	Feb 2010–Feb 2014	39	36	Open HP
Thornell et al., 2016 [15]	Sweden, Denmark	DILALA	Randomised controlled trial (9 centres)	Feb 2010–Feb 2014	43	40	Open HP
Schultz et al., 2015 [13]	Sweden, Norway	SCANDIV	Randomised controlled trial (21 centres)	Feb 2010–Jun 2014	74	70	Open or laparoscopic, HP or PRA
Vennix et al., 2015 [17]	Belgium, Italy, Netherlands	LADIES	Randomised controlled trial (42 centres)	Jul 2010–Feb 2013	45	42	Open or laparoscopic, HP or PRA
NRCSs (6)							
Samuelsson et al., 2021 [22]	Sweden	LapLav national registry	Retrospective cohort	Jan 2016–Dec 2018	173	291	Open or laparoscopic, HP or PRA
Tartaglia et al., 2019 [20]	Italy, the UK, Greece, Spain	Multicentre database	Prospective cohort	2015–2018	28	38	Laparoscopic, HP or PRA
Catry et al., 2016 [18]	France	Dual centre database	Prospective cohort	Jun 2010–Jun 2015	15	25	Open or laparoscopic, PRA
Gentile et al., 2014 [21]	Italy	Single centre database	Retrospective cohort	Jan 2009–Dec 2012	14	16	Open HP
Liang et al., 2012 [23]	USA	TEICP single centre database	Prospective cohort	May 1991–May 2010	47	41	Laparoscopic HP
Karoui et al., 2009 [19]	France	JVUH single centre database	Prospective cohort	Jan 1994–Sep 2006 (LL cohort), Jan 2000–Sep 2006 (CR cohort)	35	24	Open PRA

Critical Appraisal

**Figure 2:**
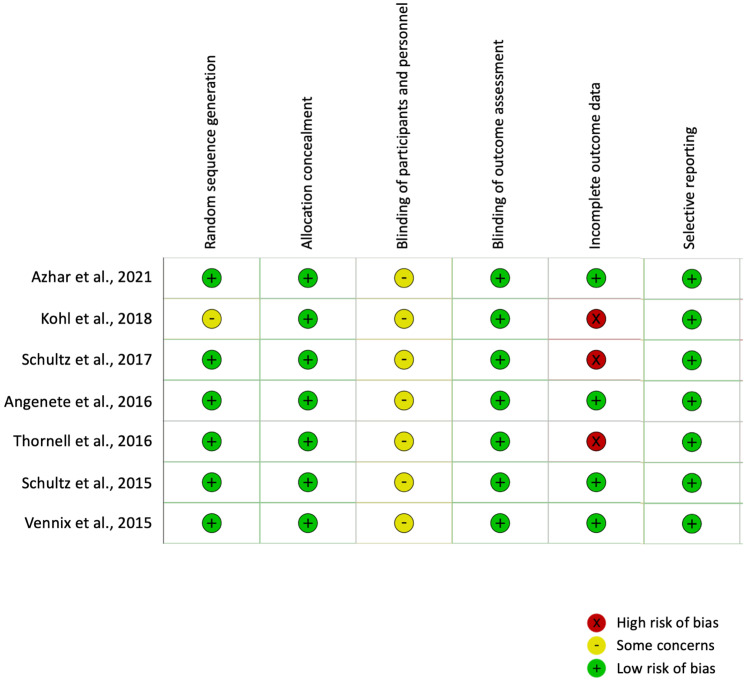
Cochrane risk-of-bias 2 assessment of randomised controlled trials included in the meta-analysis

Quality assessment scores for the seven studies [11-17] reporting data from the three RCTs and included in the meta-analysis are shown in Figure 2. Allocation to treatment groups was concealed in all studies. Given the nature of surgical interventions, blinding is usually not possible, resulting in an inherent risk of performance bias in surgical RCTs. In the current meta-analysis, objective outcome measures were used, which probably minimised the risks of differential measurement error. The risk of detection bias was low. The three studies that reported long-term outcomes were associated with high loss-to-follow-up rates (24.1% [11], 17.4% [12] and 22.9% [15]). The risk of attrition bias is higher where there are incomplete outcomes. However, this was significantly lower where short-term outcomes were reported due to a lower loss-to-follow-up rate. All seven studies were included in the meta-analysis.

Based on the MINORS tool, the quality assessment scores for the six NRCSs [18-23] ranged from 10 to 21 (Table 2). The majority of the studies had clearly stated aims, included all participants within the study period and examined relevant endpoints to the study aims. Studies with low-quality scores for these criteria were those that had non-specific aims [19,21,23], excluded participants without providing reasons (20,21), or did not specify outcomes of interest prior to analyses [23]. Only one study was prospective [20], and in another, sample size calculation was reported (40). Although there was no clear description of how outcomes were measured, thereby raising possible information bias, the chosen endpoints were objective. Three studies [19,21,23] did not report the timing of primary outcome measurement, reported loss-to-follow-up rates higher than 5% or did not report those rates. In one study [18], patients with higher ASA scores were selected for CR, which constituted a clear form of selection bias.

**Table 2:** Methodological index for non-randomised studies (MINORS) quality assessment for non-randomised surgical studies

Study	Clearly stated aim	Inclusion of consecutive patients	Prospective collection of data	Endpoints appropriate to the aim of the study	Unbiased assessment of the study endpoint	Follow-up period appropriate to the aim of the study	Loss to follow-up <5%	Prospective calculation of the study size	Adequate control group	Contemporary groups	Baseline equivalence of groups	Adequate statistical analyses	Total score (out of 24)
Samuelsson et al., 2021 [22]	2	2	0	2	2	2	2	2	2	2	1	2	21
Tartaglia et al., 2019 [20]	2	1	2	2	0	2	2	0	2	2	1	1	17
Catry et al., 2016 [18]	2	2	0	2	0	2	2	0	2	2	1	2	17
Karoui et al., 2009 [19]	1	2	0	2	0	1	0	0	2	1	2	1	12
Liang et al., 2012 [23]	1	2	0	0	0	0	1	0	2	2	2	1	11
Gentile et al., 2014 [21]	1	1	0	1	0	0	0	0	2	2	2	1	10

Patient Demographics

A total of 1,061 patients were included in this meta-analysis: 475 and 587 in the LL and the CR groups respectively; 92% of patients in both groups had Hinchey III. Overall, there were no statistically significant differences between the two treatment groups in terms of average age, gender distribution, or ASA scores.

Outcomes

**Figure 3:**
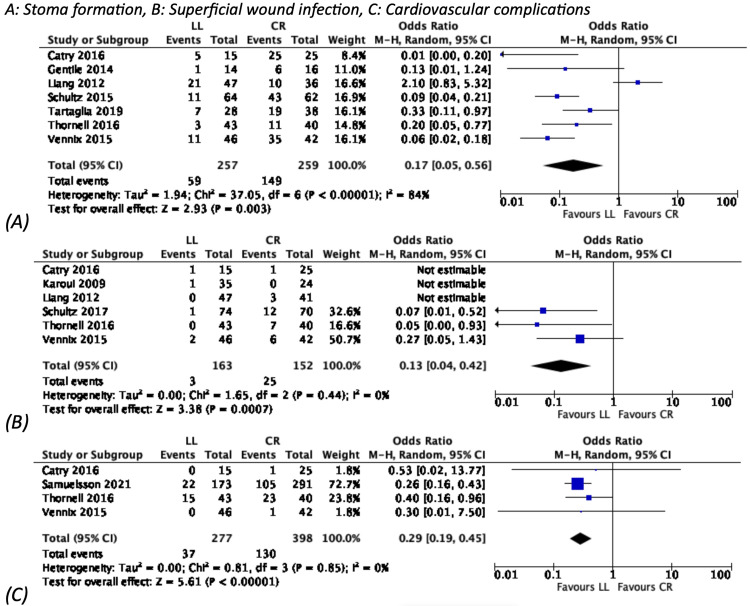
Dichotomous outcomes favouring LL CI: confidence interval; CR: colonic resection; LL: laparoscopic lavage

Compared with CR, LL was associated with lower rates of wound infection, stoma formation, and the overall need for further surgery by 77% (POR: 0.23, 95% CI: 0.07-0.74), 83% (POR: 0.17, 95% CI: 0.05-0.56), and 53% (POR: 0.47, 95% CI: 0.23-0.97) respectively (Table 3). LL was also associated with lower rates of cardiovascular complications (POR: 0.29, 95% CI: 0.19-0.45) (Figure 3). The duration of surgery and the length of hospitalisation were reduced by 54% (CWR: 0.46) and 43% (CWR: 0.57) respectively.

**Table 3:** Postoperative dichotomous outcomes for laparoscopic lavage versus resection for acute diverticulitis q value: equivalent of p-value adjusted for false discovery rate *P<0.05. **Random effects model used CI: confidence interval; POR: pooled odds ratio

Outcome	POR	95% CI	P-value	q value
30-day mortality	0.86	0.31, 2.36	0.77	0.51
90-day mortality	0.70	0.42, 1.17	0.17	0.42
1-year mortality	0.77	0.40, 1.46	0.42	0.5
Total reoperations	0.47	0.23, 0.97	0.04*	0.0004**
Unplanned reoperations	2.05	1.22, 3.42	0.006*	0.20
Unplanned readmissions	1.28	0.89, 1.84	0.18	0.39
ICU admissions	0.48	0.12, 1.93	0.30	0.0009**
Stoma formation	0.17	0.05, 0.56	0.003*	<0.001**
Recurrence	9.47	3.24, 27.67	<0.0001*	0.86
Superficial wound infection	0.23	0.07, 0.74	0.01*	0.26
Cardiovascular	0.29	0.19, 0.45	<0.0001*	0.85
Extra-abdominal	0.56	0.24, 1.31	0.18	0.03**
Intra-abdominal abscess	2.87	1.56, 5.28	0.0007*	0.62
Deep wound infection	4.12	1.71, 9.94	0.002	0.29
Peritonitis	8.92	2.71, 29.33	0.0003*	0.62

**Figure 4:**
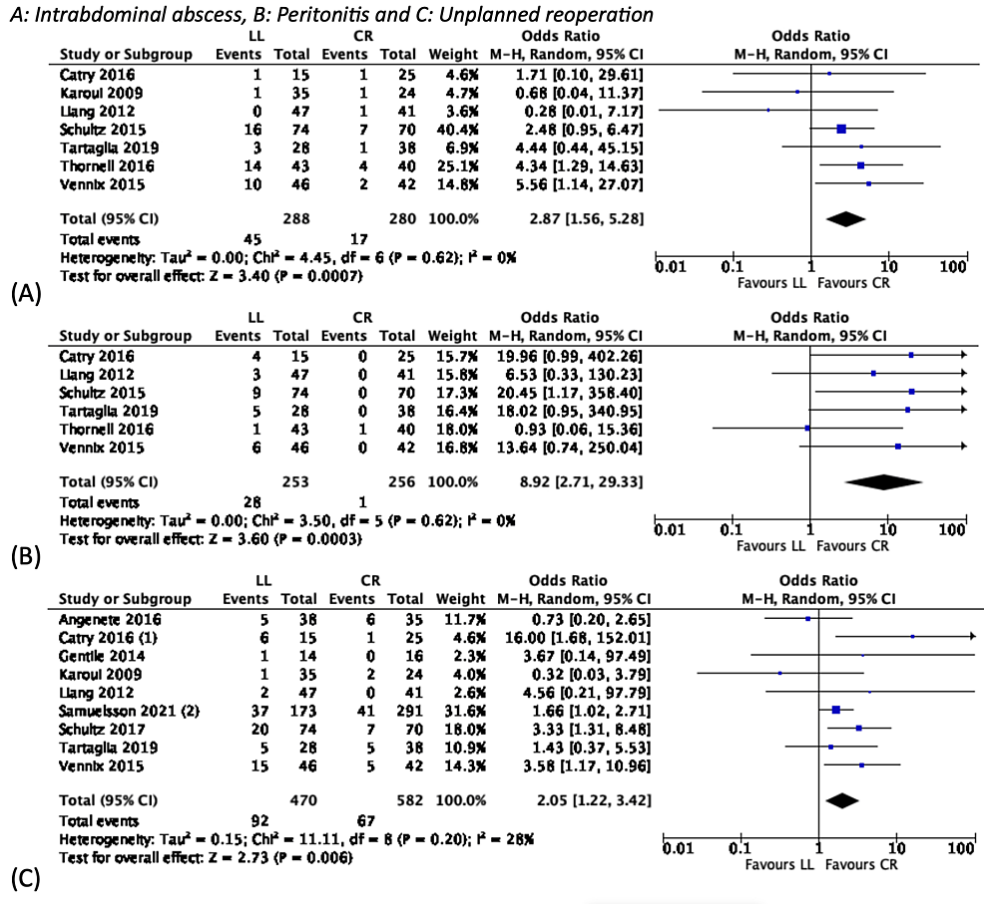
Dichotomous variables favouring CR CI: confidence interval; CR: colonic resection; LL: laparoscopic lavage

However, LL was associated with higher rates of intra-abdominal abscesses (POR: 2.87, 95% CI: 1.56-5.28), recurrence (POR: 9.47, 95% CI: 3.24-27.67), and peritonitis (POR: 8.92, 95% CI: 2.71-29.33). When an elective resection was not performed, LL was also associated with a higher risk of “unplanned” reoperation (POR: 2.05, 95% CI: 1.22-3.42) (Figure 4).

There was no statistically significant difference between the two groups in terms of mortality rates at 30 days, 90 days, and 24 months; readmission or ICU admission rates; or quality-of-life scores.

Discussion

This is the largest and most updated meta-analysis of the topic in the literature in English to date. Management of Hinchey III diverticulitis with LL remains contentious. The results of this meta-analysis support the notion that LL is a safe and feasible alternative to CR in the emergency setting. LL also conferred benefits of reduced cardiovascular and wound complications and length of hospital stay. However, the risk of unplanned reoperations in LL is higher, often with the need for subsequent CR and stoma [11,16].

Published research shows that up to 45% of colostomies are never reversed after a Hartmann procedure due to the reluctance of the surgeon and/or patient to proceed with another major procedure [25]. Patients may be too frail to undergo the perils of another anastomosis and are at a high risk of complications [26]. Furthermore, avoiding stoma formation in some patient groups such as those with progressive dementia or those dependent on care may be preferable when compared to the relatively low risk of recurrent Hinchey III diverticulitis [11,16].

One of the main drawbacks of adopting LL over CR as the management of first choice can be linked to the relatively higher risk of intra-abdominal complications and unplanned reoperations. Although the need for further surgery was significantly lower in the LL group compared to the CR group, reoperation in the CR group was mainly for stoma reversal. On the other hand, further operative measures in the LL group were largely unplanned and resulted in peritonitis and eventual CR nonetheless. In light of this, serious consideration should be given to planned elective resection under non-urgent circumstances in a patient who is adequately optimised for surgery. Performing elective procedures over emergency operations may help to reduce disease recurrence. Planned procedures also improve patients’ overall outcomes by carrying lower risks including conversion to laparotomy, stoma formation, anastomotic leak and infection. In these regards, LL may be the preferable option to avoid the risk of a permanent stoma, which by itself carries its own set of complications [25].

Another concern related to adopting the LL approach is the risk of missing a malignant lesion. Recent research suggests an 8-11% risk of concurrent malignancy in patients with acute complicated diverticulitis [27,28]. However, high-quality 64-slice CT scanning allows for a relatively accurate distinction between the two conditions [29]. Furthermore, a colonoscopy six to eight weeks after the resolution of an episode of acute complicated diverticulitis may alleviate this concern by eliminating the likelihood of missed occult malignancy, whereby an oncological resection can then be planned [3,30].

The inclusion of NRCSs might be considered a limitation of this meta-analysis due to aspects related to patient selection and study designs in those studies. For example, Samuelsson et al.'s study had over half of the patients among the NRCSs. Despite this, their study employed inverse probability treatment weighting, allowing them to achieve a better analysis of their sample size. Nonetheless, dichotomous outcomes favouring LL were predominantly gathered from the RCTs, which were well assessed on the Cochrane RoB 2 test (Figure 2). Furthermore, previous research has shown that meta-analysis of NRCSs of surgical procedures is as good as that involving RCTs [31], and subgroup analysis also showed no difference between the two sets of data. A minor limitation was the inclusion of Hinchey II cases in 3% of cases. This was due to the variations in terms of using preoperative CT findings vs. operative findings. However, it is unlikely that 3% of the data would affect the results meaningfully, especially given that the deciding factor was the need for emergent surgery based on clinical grounds. Lastly, there was variability in the definitions and reporting of outcome data and the lack of standardised protocols for LL for Hinchey III diverticulitis. For example, there was substantial variation in the volume of normal saline used for irrigation between 4 and 15 litres, the number and positions of drains, and the duration of use of antibiotics [11-22]. In addition, due to the lack of outcome data, it was also not known whether patients in the CR group who had wound complications had a laparoscopic or open CR, which may affect the outcome data for wound infection when compared to LL.

## Conclusions

LL has a role to play in the treatment of Hinchey III peritonitis in selected patients with acute diverticulitis requiring emergent surgery. There are observed benefits associated with LL in terms of duration of surgery, length of hospitalisation, wound infection, need for further surgery, extracolonic complications, and perhaps most importantly, stoma rates. The risk of missed malignancy can be eliminated by performing a routine colonoscopy six to eight weeks after the resolution of the acute episode. The risk of unplanned reoperation can be minimised by giving serious consideration to an elective resection under non-emergent circumstances. Surgeons utilising LL should be aware of the risks of recurrent diverticulitis and subsequent CR, and patients should be counselled accordingly.
